# Rapid Optical Nanomotion-Based Antibiotic Susceptibility Testing of Kombucha-Associated Acetic Acid Bacteria and *Escherichia coli*

**DOI:** 10.3390/foods15081395

**Published:** 2026-04-16

**Authors:** Meritxell Moreno Córdoba, Vjera Radonicic, Sandor Kasas, Ronnie G. Willaert

**Affiliations:** 1 Research Group Structural Biology Brussels and Alliance Research Group VUB-UGent NanoMicrobiology, Vrije Universiteit Brussel, 1050 Brussels, Belgium; meritxell.moreno.cordoba@vub.be (M.M.C.); vjera.radonicic@vub.be (V.R.); 2 Center for Psychiatric Neuroscience, Department of Psychiatry, Lausanne University Hospital, University of Lausanne, 1008 Prilly, Switzerland; sandor.kasas@unil.ch; 3 Centre Universitaire Romand de Médecine Légale (UFAM), Université de Lausanne, 1000 Lausanne, Switzerland

**Keywords:** *Komagataeibacter*, kombucha, antimicrobial resistance, optical nanomotion detection, bacterial nanocellulose, antibiotic susceptibility testing

## Abstract

Antimicrobial resistance in microorganisms associated with fermented foods is increasingly recognized, yet rapid methods to characterize antibiotic response dynamics remain limited. This study evaluates antibiotic susceptibility and physiological response patterns of kombucha-associated acetic acid bacteria and motile *Escherichia coli* using optical nanomotion detection (ONMD), a label-free technique that quantifies single-cell mechanical activity. Two cellulose-producing species (*Komagataeibacter xylinus* and *K. rhaeticus*), one non-cellulose-producing species (*K. melaceti*), and *E. coli* were exposed to ampicillin, ciprofloxacin, and chloramphenicol. Minimum inhibitory concentrations (MICs) were determined prior to time-resolved ONMD analysis. Susceptible strains exhibited progressive suppression of confined nanomotion consistent with MIC-defined susceptibility, whereas resistant profiles maintained sustained mechanical activity. Chloramphenicol initially induced persistent or increased nanomotion at 120 min; however, extending the observation to 180 min revealed delayed suppression in susceptible strains, demonstrating that bacteriostatic antibiotics require longer observation windows for accurate ONMD classification. In motile *E. coli*, ONMD revealed both intracellular nanomotion puncta and swimming trajectories, which were progressively attenuated following antibiotic exposure. These findings demonstrate that ONMD complements conventional susceptibility testing by resolving time-dependent suppression of both translational motility and intracellular nanomechanical activity at the single-cell level.

## 1. Introduction

Antimicrobial resistance (AMR) is a growing global health concern that requires coordinated efforts across clinical, environmental, agricultural, and food-production systems [1,2,3,4,5].

Fermented foods represent dynamic microbial ecosystems in which metabolically active bacteria coexist within structured communities. Kombucha, a fermented tea beverage produced by a symbiotic consortium of acetic acid bacteria (AAB) and yeasts, exemplifies such a live microbial system. One of the dominant bacterial members, *Komagataeibacter* sp., are Gram-negative, obligately aerobic, rod-shaped, good at oxidazing ethanol to acetic acid, acid-tolerant and some are capable of producing bacterial nanocellulose (BNC), forming structured biofilm-like matrices at the air–liquid interface [6,7,8]. Members of the genus belong to the family *Acetobacteraceae* and are characterised by slow growth rates, non-motile behaviour, and an optimal growth temperature typically between 25 and 30 °C. Their metabolism is strictly oxidative and they are unable to ferment sugars; instead, they incompletely oxidise alcohols and sugars to organic acids, which is the basis of their role in vinegar and kombucha production [6,7,8]. Although these bacteria are not considered primary pathogens, metagenomic analyses of fermented products have revealed the presence of antibiotic resistance genes within fermentation-associated microbiota, suggesting that AAB may contribute to a measurable food-associated resistome [9,10]. In particular, genes conferring resistance to phenicols (e.g., chloramphenicol acetyltransferase), fluoroquinolones (e.g., NorM efflux pumps), and tetracyclines have been identified in *Komagataeibacter* and related AAB, while susceptibility to β-lactams such as ampicillin appears to be generally retained [9,10]. Understanding how these organisms physiologically respond to antibiotic stress is therefore relevant for both microbial ecology and food safety monitoring. The potential risk pathway involves horizontal gene transfer resistance determinants carried by fermentation-associated bacteria can be transferred via conjugative plasmids, transposons, or integrons to commensal or pathogenic organisms within the human gastrointestinal tract upon ingestion of live fermented products [9,11]. Because kombucha is consumed mostly unpasteurised and contains viable bacteria at the point of consumption, it provides a direct route for introducing resistant AAB into the gut microbiome, where they may coexist with clinically relevant species. This ecological concern is amplified by the fact that many resistance genes identified in food-associated microbiota, including those encoding efflux pumps and ribosomal protection proteins, are located on mobile genetic elements with broad host range [11,12,13]. Rapid phenotypic characterisation of antibiotic responses in these organisms is therefore important not only for fermentation quality control but also for assessing the potential contribution of fermented foods to the dissemination of antimicrobial resistance.

Resistance traits in AAB have been reported for several antibiotics, including trimethoprim, erythromycin, ciprofloxacin, and chloramphenicol, with certain strains displaying multidrug resistance profiles [9,10]. As Gram-negative bacteria, *Komagataeibacter* species possess structural and physiological features that can influence antibiotic susceptibility, such as an outer membrane barrier and active efflux systems [11,12,13]. However, conventional antimicrobial susceptibility testing relies on growth-based endpoints, such as minimum inhibitory concentration (MIC) determination, which provide limited insight into early physiological responses or sublethal stress dynamics. Growth inhibition does not necessarily reflect immediate metabolic disruption, and different mechanisms of action may not be distinguishable within short observation windows [14]. 

Antibacterial agents can be classified according to their mechanisms of action, including inhibitors of cell wall synthesis (e.g., ampicillin), inhibitors of protein synthesis (e.g., chloramphenicol, erythomycin), folate synthesis inhibitor (e.g., trimetrhoprim), and inhibitors of nucleic acid synthesis through interference with DNA replication or transcription (e.g., ciprofloxacin) [1,15,16,17]. These mechanisms ultimately perturb essential cellular processes such as peptidoglycan assembly, ribosomal function, or DNA topology. Bacterial resistance strategies, including reduced drug uptake, target modification, enzymatic inactivation, and active efflux, modulate the extent to which these perturbations translate into metabolic arrest [1,16,17,18]. A method capable of resolving these physiological perturbations in real time at the single-cell level would provide mechanistic insight beyond binary growth/no-growth endpoints.

Nanoscale mechanical fluctuations, referred to as nanomotion, are a universal feature of metabolically active cells and arise from intracellular processes [19,20,21]. Nanomotion was successfully detected in virtually all living organisms on Earth including bacteria, yeast, vegetal and mammalian cells [19,20,21,22,23,24,25,26]. Among bacteria it was demonstrated in motile, non-motile, Gram-positive, Gram-negative, and spores. Nanomotion persists as long as the organism is alive and diminishes when cellular metabolism collapses and disappears upon loss of viability. Nanomotion has been detected using atomic force microscopy (AFM)-based cantilever systems [19,22,23], suspended membrane resonators called nanodrums [27], optical tweezers [28], and more recently through optical nanomotion detection (ONMD), which quantifies frame-to-frame displacement of single cells using standard optical microscopy and video analysis [24,25,26].

Willaert et al. [23] first demonstrated that a standard optical microscope equipped with a video camera can detect nanometric-scale oscillations of non-attached single yeast cells, establishing ONMD as a viable alternative to AFM-based methods. Using microwell-based ONMD, Radonicic et al. [24] subsequently showed that the susceptibility of *Candida albicans* to caspofungin and fluconazole could be assessed within 10 min of antifungal exposure. Villalba et al. [25] extended the technique to bacteria, demonstrating single-cell antibiotic response testing for motile *E. coli* (ampicillin, doxycycline), non-motile Gram-positive *Staphylococcus aureus* (vancomycin), and slow-growing *Mycobacterium smegmatis* (streptomycin), with results obtained within 2–5 h depending on the species. A simplified diff-image protocol was subsequently validated for *S. aureus*, *E. coli*, *C. albicans*, and *Saccharomyces cerevisiae* [26]. However, all published ONMD studies to date have focused on well-characterised clinical or model organisms with established susceptibility breakpoints, relatively fast growth rates, and no extracellular matrix production. No ONMD study has yet addressed non-clinical, environmental, or food-associated bacteria, nor organisms with intrinsically low nanomotion signal. ONMD is label-free, attachment-free, and sensitive to rapid physiological changes, allowing detection of antibiotic-induced metabolic perturbations within minutes to hours. Importantly, nanomotion remains measurable in viable but non-proliferating cells, enabling insight into antibiotic-induced physiological changes in short-term experiments [24].

To date, ONMD has been applied exclusively to clinical isolates, including Gram-negative and Gram-positive bacteria, mycobacteria, and yeast [23,24,25,26]. Acetic acid bacteria associated with fermented foods have not been investigated, despite their relevance to the food-associated resistome. *Komagataeibacter* species present distinct challenges for ONMD: they are non-motile, slow-growing, and exhibit intrinsically low baseline nanomotion, limiting the dynamic range for detecting antibiotic-induced changes. In addition, two of the three species tested here produce bacterial nanocellulose, which may influence cell behaviour within the analysis chamber. Furthermore, no standardised clinical breakpoints (CLSI/EUCAST) exist for these organisms, requiring empirical susceptibility classification. These characteristics make *Komagataeibacter* a methodologically informative test case for extending ONMD beyond clinical microbiology toward food-associated microbiota.

In this study, we investigate the antibiotic response dynamics of two cellulose-producing AAB involved in kombucha fermentation, *Komagataeibacter rhaeticus* and *K. xylinus*, together with a non-cellulose-producing strain, *K. melaceti*. *E. coli* was included as a reference organism and potential contaminant. By combining conventional MIC determination with time-resolved single-cell ONMD analysis, we assess whether antibiotic-induced perturbations in cellular physiology can be resolved through nanomotion signatures and whether cellulose-producing and non-producing strains exhibit distinct mechanical response profiles. This mechanistic approach aims to extend antimicrobial susceptibility assessment in fermentation-associated microbiota beyond growth-based endpoints toward dynamic single-cell physiological characterization. By establishing whether ONMD can rapidly discriminate between susceptible and resistant phenotypes in these non-clinical organisms, this work provides a basis for applying label-free nanomotion sensing as a screening tool to evaluate antibiotic resistance in fermented food microbiota, thereby supporting risk assessment of antimicrobial resistance dissemination through the food chain.

## 2. Materials and Methods

### 2.1. Culture Conditions

The strains *K. xylinus* LMG 1518, *K. rhaeticus* LMG 22126, *K. melaceti* LMG 31303 and *E. coli* LMG 2092 deriving from BCCM/LMG Bacteria Collection (Ghent, Belgium) were revived following the BCCM instructions. All strains were stored in the appropriate medium supplemented with 25% (*v*/*v*) glycerol (Sigma-Aldrich, Overijse, Belgium) as cryoprotectant, at −80 °C. *E. coli* was stored in Nutrient Broth medium (Sigma-Aldrich, Overijse, Belgium), *K. melaceti* was stored in BCCM/LMG 239 medium (40 g/L glucose, 10 g/L yeast extract, 10 g/L peptone, 3.38 g/L Na_2_HPO_4_ · 12H_2_O, 1.5 g/L citric acid, 20 mL/L ethanol, 10 mL/L citric acid) (all chemicals from Sigma-Aldrich, Overijse, Belgium), *K. rhaeticus* and *K. xylinus* were stored in BCCM/LMG 129 medium (50 g/L glucose, 5 g/L yeast extract). All *Komagataeibacter* strains were grown at 30 °C and *E. coli* at 37 °C.

Prior to their utilization, the cells were cultured by an overnight inoculation of 150 mL of the appropriate medium with a colony from an agar medium plate (medium containing 15 g/L agar) in 300 mL Erlenmeyer flask with ridges at their optimal growing temperature and 180 revolutions per minute (rpm) in an Incubator shaker (Innova 4400, New Brunswick, Edison, NJ, USA). The cultures were further diluted by adjusting the optical density (OD_600_ value) to 0.02 for optimal visualization. Under these shaken culture conditions, *Komagataeibacter* cells grew planktonically and were not embedded in a nanocellulose matrix at the time of ONMD measurement; antibiotic diffusion to individual cells was therefore not impeded by extracellular cellulose.

### 2.2. MIC Determination

Prior to assessing the nanomotion changes detected in the targeted microorganisms because of the antibiotics a quantitative minimum inhibitory concentration (MIC) test was performed. Antibiotic gradient test strips (Liofilchem^®^, Roseto degli Abruzzi, Italy) of ampicillin, chloramphenicol and ciprofloxacin were used on confluent lawn of growth agar plates following the manufacturer’s instructions. The selection of antibiotics was based on previously published susceptibility studies in *Komagataeibacter* species and related acetic acid bacteria [9,10]. Tests were performed on BCCM/LMG 129 agar medium for the *Komagataeibacter* spp. and on nutrient broth-agar medium for *E. coli*. The strips are impregnated with a gradient concentration of the antibiotic which is indicated on the strip. After the indicated incubation time at the optimal temperature, a symmetrical inhibition ellipse is formed along the strip indicating the MIC of that antibiotic for that bacterial strain.

### 2.3. Antibiotic Solutions Preparation

For each antibiotic–bacteria combination four concentrations were prepared: a control without antibiotic, the MIC, a higher antibiotic concentration and an even higher one that corresponds to the highest concentration of the MIC strips used before. Concentrations above the MIC were included to evaluate dose-dependent nanomotion suppression and to assess whether suprainhibitory levels accelerate signal decay. Ciprofloxacin required the addition of HCl to a final concentration of 0.01 N to fully dissolve it. Antibiotic stock solutions were stored at −20 °C and fresh working dilutions were prepared immediately before each experiment. At the final concentrations used in the ONMD assays, the HCl contributed by the ciprofloxacin stock was diluted to negligible levels (<0.001 N in the bacterial culture), well below concentrations known to affect bacterial physiology or nanomotion. In addition, because all ciprofloxacin conditions, including the untreated control, received the same solvent background, any residual solvent effect was controlled for in the experimental comparison.

### 2.4. ONMD Analysis Chamber Setup

To entrap the cells several analysis chambers were prepared by cutting a 6 mm diameter hole in a double-coated adhesive tape (Nitto Denko Corporation, Osaka, Japan) of 10 µm thickness as described previously [25]. The tape was stuck to a microscope glass slide and the hole left in the middle was impregnated with biocompatible fluorinated oil (Fluo-Oil; Darwin Microfluidics, Paris, France) to avoid cell adhesion to the glass. From the bacterial solution prepared before 0.8 µL was added and the hole was then covered with a cover Topas COC (cyclic olefin copolymer) film (glass transition temperature, Tg = 142 °C, thickness 175 µm; Microfluidic ChipShop GmbH, Jena, Germany) which was also covered in oil to avoid adhesion of the cells to the surface [25]. The biocompatibility of fluorinated oil with bacterial cells was previously validated: *E. coli* and *S. aureus* incubated in oil for 5 h showed no effect on nanomotion signal or cell viability [25,26].

### 2.5. Optical Nanomotion Detection

To enable ONMD through bright-field microscopy, the microfluidic chip setup was mounted on an inverted Nikon Eclipse Ti2 epifluorescence microscope (Nikon, Tokyo, Japan). Between three to five fields of views were recorded, aiming to collect more than 20 cells per time point. Four time points were collected: before applying the antibiotic to the cells, 10 min after application and incubation, one hour incubation and the last one after two hours of incubation. These time points were selected to span both rapid and delayed antibiotic effects on a logarithmic-like scale: the 10 min time point was included because prior ONMD work demonstrated that antifungal susceptibility of *C. albicans* could be detected within 10 min [24], and we aimed to test whether similarly rapid effects occur in bacteria; 60 and 120 min capture the progressive physiological perturbations typically observed for bactericidal agents; an additional 180 min time point was included for *Komagataeibacter* spp. to detect delayed responses from bacteriostatic antibiotics such as chloramphenicol. Uniform 30 min intervals were not used because the experimental setup required recording 3–5 fields of view per condition at each time point across multiple antibiotic concentrations, making finer temporal resolution impractical within a single experiment while maintaining sufficient replicates per condition. All videos lasted 17 s with a frame rate of 20 fps and were recorded using an EMCCD camera (Andor, Oxford Instruments plc, Abingdon, UK) and a 40× objective (1 pixel corresponded to 0.16 µm) in bright-field mode. Three videos per condition and time point were analysed; each video integrates the pixel intensity changes in all cells present in the field of view, but constitutes a single statistical observation (*n* = 1), as the cells within one video share the same experimental conditions and are not statistically independent. Each strain–antibiotic combination was tested in a single experimental run; the three to five fields of view per condition therefore represent technical replicates from the same microfluidic chip preparation and culture. Independent biological replicates (separate experiments from independent cultures) were not performed. This is acknowledged as a limitation of the current study.

The resulting videos were processed using a cross-correlation ONMD algorithm (MATLAB R2024a, MathWorks) by calculating the x-y displacement of individual cells for each frame as described previously [23]. The trajectories of the tracked cells and the average displacement were then saved. The velocity (µm/s) of all the tracked cells through all the time points were represented as violin and box-and-whisker (10th to 90th percentile) plots for each antibiotic–bacteria combination (Prism10, GraphPad). Optical changing-pixel images were also collected, indicating the pixels that changed the most in red in contrast to the ones that changed the least in blue.

Considering *E. coli* is a motile bacterium, a different approach to analyse the videos was taken since cells moved in and out the FOV during the recording of the movies, which can result in over- or underestimated nanomotion velocities (as measured using the cross-correlation algorithm). Three videos of every time point and condition were analysed using the home-developed Python software 3.7 which enables frame-to-frame displacement analysis and nanomotion quantification from time-lapse microscopy videos. The program generates a differential (“Diff”) image by subtracting each frame of the movie from a subsequent frame separated by a defined number of frames (deltaF) and calculating the absolute pixel intensity differences [26]. These differences are accumulated over the entire sequence to produce a motion-highlight image in which brighter regions indicate frequent or large displacements, while static areas remain dark; the resulting quantitative output corresponds to the total pixel intensity change over time. Both algorithms quantify cellular mechanical activity through complementary metrics, single-cell displacement velocity and integrated pixel intensity change, respectively; and within each organism, identical acquisition and analysis parameters are applied to treated and control conditions, ensuring internal comparability.

### 2.6. Statistical Analysis

Statistical analyses were performed using GraphPad Prism 10.6.1 (GraphPad Software, San Diego, CA, USA). For each detected cell, the instantaneous frame-to-frame velocities recorded during a single video acquisition were averaged to obtain one mean velocity value per cell, which served as the unit of statistical analysis. This approach avoids pseudoreplication arising from treating multiple temporally correlated velocity measurements within a single cell as independent observations.

For all *Komagataeibacter* spp. experiments, differences between the pre-treatment baseline (t = 0 min) and each post-treatment time point were evaluated using the Mann–Whitney U test (two-tailed), a non-parametric test for unpaired comparisons selected due to the expected non-normal distribution of single-cell nanomotion data. Comparisons are unpaired because different cells are detected and tracked at each time point. The baseline (t = 0) comprises pooled cell means from all concentration conditions measured prior to antibiotic exposure. The sample size (*n*) represents the number of individual cells analysed per condition and time point, ranging from 20 to 95 cells. Statistical significance is indicated as *p* < 0.05 (*), *p* < 0.01 (**), *p* < 0.001 (***), and *p* < 0.0001 (****); ns indicates not significant. Because each experimental condition was performed as a single experiment without independent biological replicates, the reported *p*-values reflect within-experiment variability across individual cells and should be interpreted accordingly.

For all *E. coli* experiments, differences between antibiotic concentrations for each time point were evaluated using the Welch’s *t*-test (two-tailed, unequal variances assumed), as the Mann–Whitney U test cannot achieve *p* < 0.05 with *n* = 3 per group. The sample size (*n*) represents the total pixel change for all cells present in one video, analysed per condition and time point (*n* = 3). Statistical significance is indicated as above.

## 3. Results

### 3.1. MIC Determination and Definition of Exposure Conditions

Minimum inhibitory concentrations (MICs) determined by gradient strip assays are summarized in Table 1. *E. coli* exhibited clearly defined inhibition ellipses for all three antibiotics, allowing unambiguous MIC determination. In contrast, the *Komagataeibacter* strains displayed heterogeneous susceptibility profiles.

Ampicillin produced measurable inhibition zones in all tested *Komagataeibacter* strains, although MIC values varied considerably (0.75–24 µg/mL) (Table 1). Resistance to chloramphenicol was observed in *K. xylinus* and *K. melaceti*, as no inhibition endpoint was detected within the tested concentration range. *K. melaceti* additionally exhibited resistance to ciprofloxacin. It should be noted that no CLSI or EUCAST clinical breakpoints are currently defined for *Komagataeibacter* species; the susceptibility classifications reported here are therefore based on the MIC values obtained from gradient strip assays and should be interpreted within this experimental context rather than as clinical designations.

The MIC values were subsequently used to define ONMD exposure concentrations, including sublethal (MIC), supra inhibitory, and maximal strip concentrations, enabling analysis of concentration-dependent nanomotion dynamics. These MIC-defined susceptibility profiles were used as reference points for interpreting nanomotion suppression dynamics.

### 3.2. Antibiotic-Induced Nanomotion Dynamics in Komagataeibacter Species

**Figure 1:**
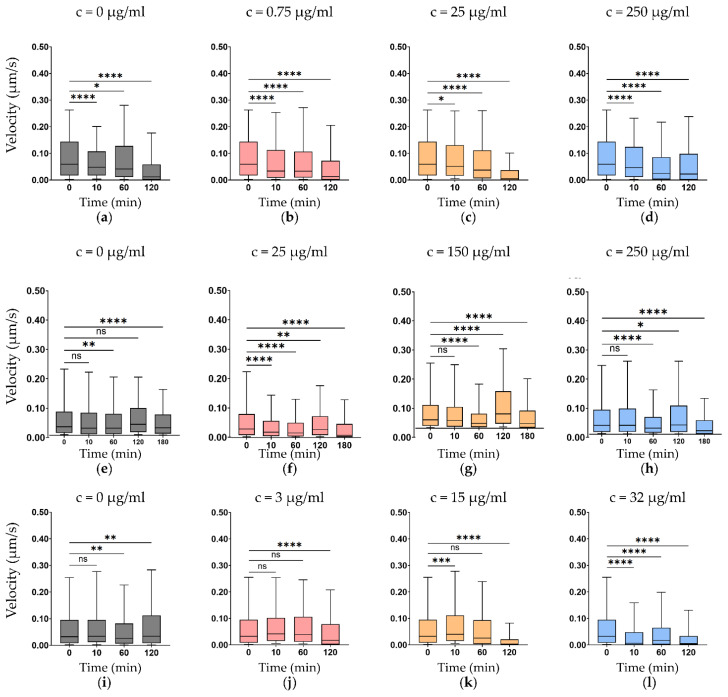
Nanomotion response of *K. xylinus* exposed to increasing concentrations over time: (**a**–**d**) ampicillin at 0, 0.75 (MIC), 25, and 250 µg/mL, (**e**–**h**) chloramphenicol at 0, 25, 150, and 250 µg/mL, and (**i**–**l**) ciprofloxacin at 0, 3 (MIC), 15, and 32 µg/mL. Boxplots show the distribution of cell velocity (µm/s), used as an indicator of cellular nanomotion, measured before treatment 0, and 10, 60, and 120 min after antibiotic exposure (and 180 min for chloramphenicol). Boxes represent the interquartile range with the median indicated by a horizontal line; whiskers show 10 and 90 percentile values. Statistical comparisons use cell-mean velocities (*n* = 20–95 cells per condition and timepoint) versus a pooled baseline (*n* = 240 cells at t = 0). Mann–Whitney U test: *p* < 0.05 (*), *p* < 0.01 (**), *p* < 0.001 (***), and *p* < 0.0001 (****); ns not significant.

**Figure 2:**
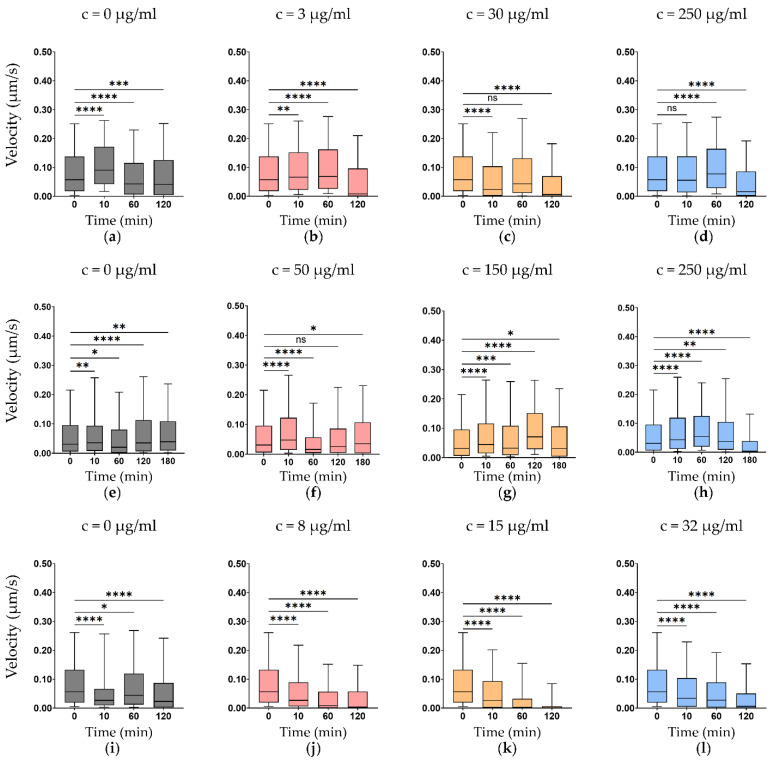
Nanomotion response of *K. rhaeticus* exposed to increasing concentrations over time: (**a**–**d**) ampicillin at 0, 3 (MIC), 30, and 250 µg/mL, (**e**–**h**) chloramphenicol at 0, 50 (MIC), 150, and 250 µg/mL, and (**i**–**l**) ciprofloxacin at 0, 8 (MIC), 15, and 32 µg/mL. Boxplots show the distribution of cell velocity (µm/s), used as an indicator of cellular nanomotion, measured before treatment 0, and 10, 60, and 120 min after antibiotic exposure (and 180 min for chloramphenicol). Boxes represent the interquartile range with the median indicated by a horizontal line; whiskers show 10 and 90 percentile values. Statistical comparisons use cell-mean velocities (*n* = 20–95 cells per condition and timepoint) versus a pooled baseline (*n* = 240 cells at t = 0). Mann–Whitney U test: *p* < 0.05 (*), *p* < 0.01 (**), *p* < 0.001 (***), and *p* < 0.0001 (****); ns not significant.

**Figure 3:**
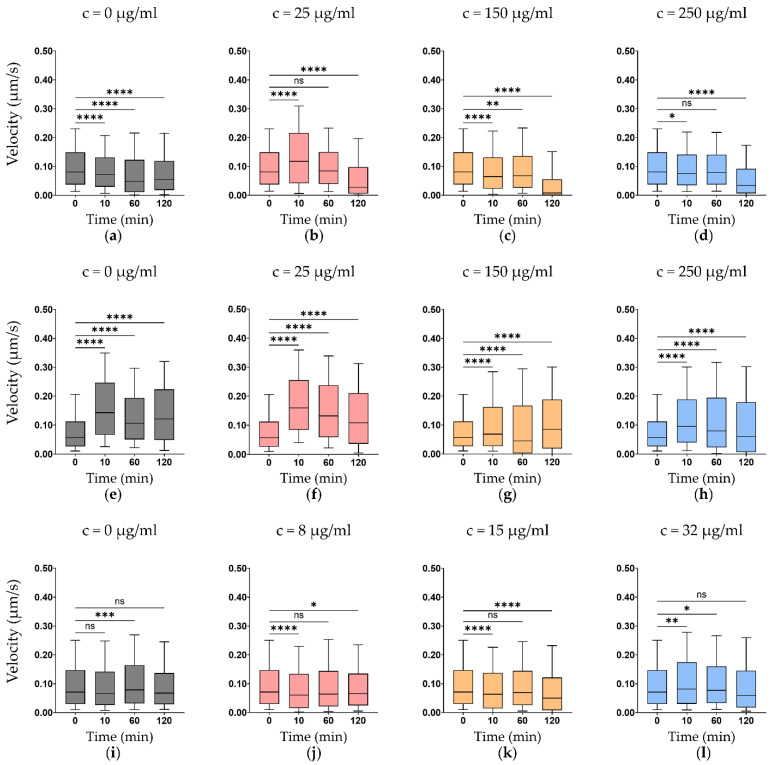
Nanomotion response of *K. melaceti* exposed to increasing concentrations over time: (**a**–**d**) ampicillin at 0, 25 (MIC), 150, and 250 µg/mL, (**e**–**h**) chloramphenicol at 0, 25, 150, and 250 µg/mL (note: all tested concentrations are sub-MIC, as the MIC for chloramphenicol was > 256 µg/mL; Table 1), and (**i**–**l**) ciprofloxacin at 0, 8, 15, and 32 µg/mL. Boxplots show the distribution of cell velocity (µm/s), used as an indicator of cellular nanomotion, measured before treatment 0, and 10, 60, and 120 min after antibiotic exposure. Boxes represent the interquartile range with the median indicated by a horizontal line; whiskers show 10 and 90 percentile values. Statistical comparisons use cell-mean velocities (*n* = 20–95 cells per condition and timepoint) versus a pooled baseline (*n* = 240 cells at t = 0). Mann–Whitney U test: *p* < 0.05 (*), *p* < 0.01 (**), *p* < 0.001 (***), and *p* < 0.0001 (****); ns not significant.

Exposure to antibiotics resulted in concentration- and time-dependent alterations in nanomotion across all *Komagataeibacter* strains (Figure 1, Figure 2 and Figure 3). In susceptible conditions, ampicillin and ciprofloxacin induced progressive reductions in median cell velocity between 0 min (before treatment) and 120 min. With ampicillin treatment, median velocity decreased between 26.8–63.8% (Figure 1a–d, Figure 2a–d and Figure 3a–d), and with ciprofloxacin between 17.0–70.1% (Figure 1i–l, Figure 2i–l and Figure 3i–l). Suppression was more pronounced at concentrations at or above the MIC, consistent with effective physiological perturbation.

Under resistant conditions, nanomotion signals remained detectable throughout the 120-min observation window, indicating preserved metabolic activity. Chloramphenicol treatment initially resulted in increased nanomotion at 120 min across all *Komagataeibacter* strains (Figure 1e–h, Figure 2e–h and Figure 3e–h), including the resistant *K. xylinus* and *K. melaceti* (up to +63.3%), notably, for *K. xylinus* and *K. melaceti*, all tested chloramphenicol concentrations (25, 150, and 250 µg/mL) were sub-MIC, as both strains had MIC values > 256 µg/mL (Table 1), as well as the susceptible *K. rhaeticus*, consistent with the bacteriostatic mechanism of action of chloramphenicol, which inhibits protein synthesis without directly killing cells [29,30,31]. However, when the observation window was extended to 180 min, chloramphenicol-treated *K. xylinus* showed a decline in nanomotion (−20.4 to −34.9%), and *K. rhaeticus* exhibited a concentration-dependent response, with nanomotion still increasing at the MIC and supra-MICs (+7.3% and +7.4%) but declining at the maximum concentration (−43.8%). These delayed responses are consistent with the bacteriostatic mechanism of chloramphenicol, where growth inhibition precedes suppression of metabolic activity. *K. melaceti* proved resistant to ciprofloxacin in both the MIC test and the ONMD test, with a reduction in nanomotion of only 4.8–18.7% after 120 min. In comparison, the susceptible strains of *Komagataeibacter* showed a more than twofold decline in nanomotion: 17.0–57.5% for *K. xylinus* and 48.3–70.1% for *K. rhaeticus*.

A systematic comparison of MIC-based susceptibility classification and ONMD-derived nanomotion responses is presented in Table 2. For all *Komagataeibacter* strains, ONMD classifications were largely concordant with MIC-defined susceptibility profiles: susceptible conditions generally showed nanomotion decline exceeding 40%, resistant conditions showed preserved or increased nanomotion, and intermediate responses fell within the 20–40% range. Chloramphenicol initially induced increased nanomotion across all *Komagataeibacter* strains at 120 min. However, extending the observation to 180 min revealed a delayed suppression of nanomotion in *K. xylinus* and *K. rhaeticus*, consistent with a delayed response: an intermediate delayed response in *K. xylinus* (suppression 20–35%) and a delayed susceptible response in *K. rhaeticus* (suppression reaching > 40% at the highest concentration). *K. melaceti* maintained increased nanomotion throughout, consistent with its MIC-defined resistant phenotype. *K. melaceti* exhibited only a modest nanomotion decline following ciprofloxacin exposure (4.8–18.7%), substantially smaller than the suppression observed in susceptible *Komagataeibacter* strains. This weak physiological perturbation is consistent with the MIC-defined resistant phenotype (>32 µg/mL).

### 3.3. Motility-Dependent and Intracellular Nanomotion Patterns in Escherichia coli

**Figure 4:**
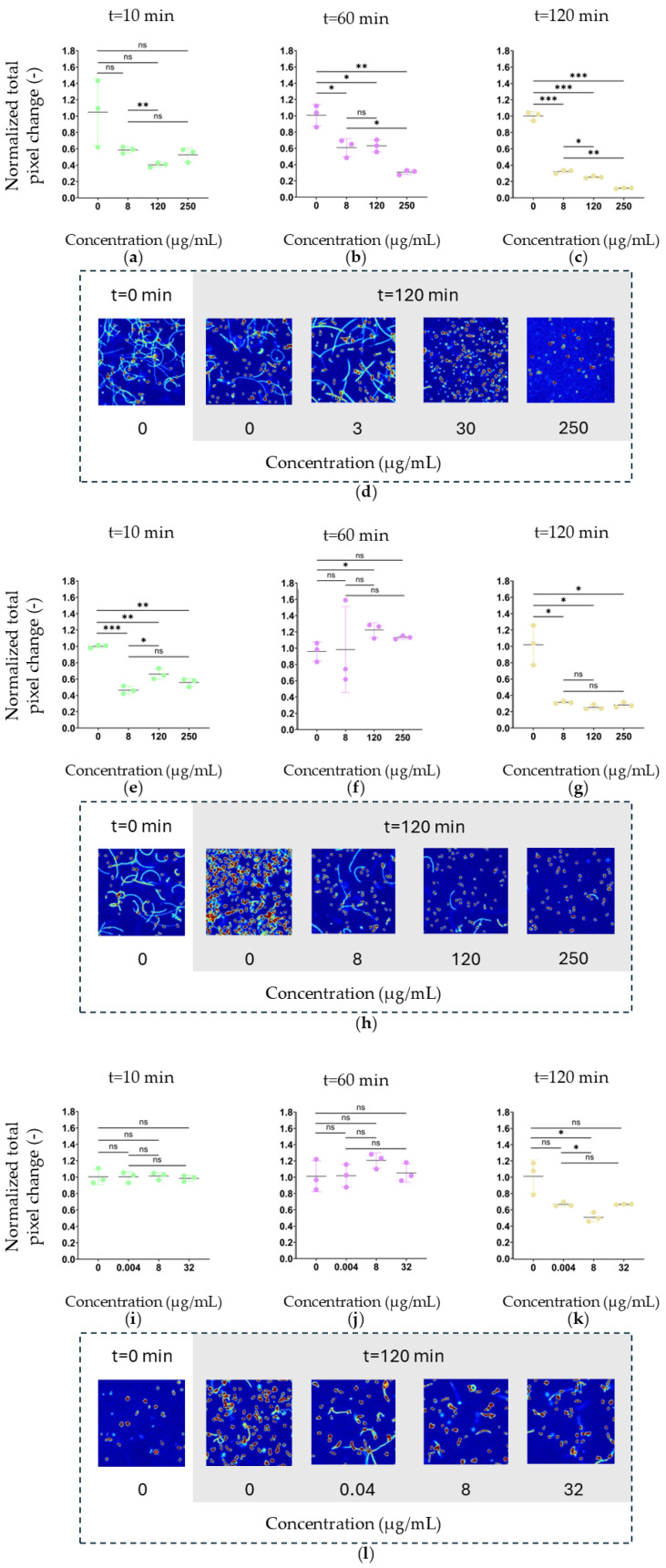
Nanomotion-derived pixel variation in *E. coli* exposed to increasing (**a**–**d**) ampicillin, (**e**–**h**) chloramphenicol and (**i**–**l**) ciprofloxacin concentrations. Total pixel change measured at 10 min (green), 60 min (pink), and 120 min (yellow) after antibiotic exposure, plotted against concentration. Each dot represents the total pixel change of a single video, and horizontal lines indicate mean values. *n* = 3 videos per condition and timepoint. (**d**,**h**,**l**) Representative optical changing pixel heatmaps acquired at t = 0 and t = 120 min for the corresponding concentrations. Welch’s *t*-test: *p* < 0.05 (*), *p* < 0.01 (**), and *p* < 0.001 (***); ns not significant.

In contrast to *Komagataeibacter* species, *E. coli* exhibited a composite mechanical signal in optical changing pixel (OCP) heatmaps (Figure 4d,h,l) consisting of both discrete single-cell nanomotion puncta and elongated cyan or bright linear traces corresponding to swimming trajectories during the 17-s acquisition period. Signal intensity is displayed according to the color scale, with red indicating higher nanomotion activity and blue indicating lower activity. The punctate signals reflect confined intracellular nanomotion, whereas the linear traces represent active translational motility. At baseline (before antibiotic exposure), motility-associated trajectories were prominent and frequently overlapped with discrete nanomotion puncta, indicating that both motion modalities contributed to the overall pixel variation. Following antibiotic exposure, motility-associated traces diminished in a concentration- and time-dependent manner. Ampicillin exposure led to rapid reduction in nanomotion signal at concentrations at or above the MIC (Figure 4). Chloramphenicol (Figure 4h) and ciprofloxacin (Figure 4l) similarly attenuated motility-associated signals, with progressive shortening and disappearance of swimming traces over time.

In all cases, after 120 min the median velocity decreased, comparing the non-treated experiment with the highest concentration, 88.3% for ampicillin (Figure 4c), 72.3% for chloramphenicol (Figure 4g), and 33.8% for ciprofloxacin (Figure 4k). These endpoint reductions are summarised in Table 2. The temporal progression of nanomotion suppression differed markedly between antibiotics. For ampicillin, no significant reductions in total pixel change were observed at 10 min, but by 60 min all concentrations showed significant decreases, and by 120 min suppression was highly significant across all concentrations (−49.7 to −88.3%). Chloramphenicol exhibited a distinct biphasic temporal profile: an early significant reduction at 10 min, followed by partial recovery or increase at 60 min, and renewed suppression at 120 min (down to −75.2%). This biphasic pattern is consistent with an initial bacteriostatic shock followed by transient metabolic compensation before sustained protein synthesis inhibition takes effect. Ciprofloxacin showed limited effects, with no significant changes at 10 or 60 min; only at 120 min did the intermediate concentration (8 µg/mL) reach statistical significance (−49.5%), while other concentrations showed non-significant reductions (Table 2). Statistical comparisons were performed using Welch’s *t*-test (*n* = 3 videos per condition and timepoint).

At higher antibiotic concentrations, OCP maps transitioned from trajectory-dominated patterns to isolated puncta and eventually to low-level diffuse signals. This progression suggests that translational motility and confined intracellular nanomotion are differentially suppressed under antibiotic stress.

These observations demonstrate that ONMD captures distinct modes of bacterial motion, active swimming and intracellular nanomechanical fluctuations, and resolves their differential suppression under antibiotic stress.

## 4. Discussion

The present study demonstrates that ONMD enables time-resolved characterization of antibiotic-induced physiological perturbations in kombucha-associated acetic acid bacteria and *E. coli* at the single-cell level. Combining MIC testing with nanomotion analysis allowed antibiotic susceptibility to be measured through both growth inhibition and mechanical signals indicating metabolic disruption.

The three antibiotics selected, ampicillin, chloramphenicol, and ciprofloxacin, target distinct cellular processes (cell wall synthesis, protein synthesis, and DNA replication, respectively), and the ONMD susceptibility profiles obtained here were consistent with those previously reported for *Komagataeibacter* spp. (Table 2) [9,10]. A mechanism-dependent temporal discrepancy was observed for chloramphenicol. At 120 min, all *Komagataeibacter* strains exhibited increased or preserved nanomotion, suggesting a resistant ONMD pattern. However, extending the observation window to 180 min revealed delayed nanomotion suppression in *K. xylinus* (−20.4 to −34.9%) and a concentration-dependent response in *K. rhaeticus*, where suppression reached −43.8% only at the highest concentration. This delayed response likely reflects the bacteriostatic mode of action of chloramphenicol, which inhibits protein synthesis without immediately abolishing basal mechanical activity. For bacteriostatic antibiotics, the standard 120 min ONMD window may therefore underestimate susceptibility, and an extended observation period is recommended. Notably, *K. xylinus* was classified as resistant by MIC (>256 µg/mL) but showed a delayed intermediate ONMD response at 180 min. This suggests that ONMD can detect physiological perturbation not captured by growth-based assays, possibly because chloramphenicol perturbs metabolic activity in *K. xylinus* even when growth inhibition is absent at standard MIC testing concentrations. However, the observed suppression values for *K. xylinus* at 180 min (−20.4 to −34.9%) fall within the intermediate range (20–40%) rather than exceeding the >40% threshold for full susceptibility. The “Intermediate (delayed)” designation thus reflects a trend toward suppression that has not yet reached the susceptibility threshold; future extended-observation studies may clarify whether suppression progresses beyond 40% at later time points. Interestingly, ciprofloxacin also induced a modest reduction in nanomotion in the resistant strain *K. melaceti*. This suggests that ONMD can detect early physiological perturbations even in resistant bacteria, although the perturbations are insufficient to fully suppress metabolic activity. ONMD thus provides insight into sublethal stress responses not captured by growth-based MIC measurements, consistent with previous studies in which nanomotion suppression as low as 14.5% (*M. smegmatis* exposed to streptomycin) were reported as statistically significant and biologically meaningful [26], was reported as biologically meaningful [26].

The observed resistance and susceptibility profiles are consistent with known genetic determinants in acetic acid bacteria, including efflux pumps, enzymatic inactivation, and porin-mediated drug entry [9,10].

In *Komagataeibacter* species, antibiotic exposure resulted in progressive suppression of confined single-cell nanomotion under susceptible conditions, whereas resistant profiles maintained sustained mechanical activity (Figure 1, Figure 2 and Figure 3) consistent with MIC-defined susceptibility and previous studies [9,10]. Chloramphenicol produced variable nanomotion responses, sometimes reduced, sometimes increased, suggesting that ONMD captures metabolic activity independently of proliferation [29,30,31]. The observation that chloramphenicol initially increased nanomotion at 120 min in both susceptible and resistant strains warrants mechanistic consideration. Chloramphenicol binds the 50S ribosomal subunit and blocks peptidyl transferase activity, halting elongation of nascent polypeptide chains. However, this does not immediately abolish cellular energy metabolism; rather, cells remain energised and may redirect metabolic flux toward stress-compensatory pathways. Translational arrest triggers the stringent response via accumulation of (p) ppGpp [31], which reprograms metabolism and increases ATP turnover, and may activate envelope stress pathways leading to increased membrane remodelling—processes that generate mechanical fluctuations detectable by ONMD. The biphasic response—initial hyperactivity followed by eventual suppression at 180 min—is thus consistent with an acute stress-compensation phase giving way to progressive metabolic exhaustion as essential protein pools become depleted. As a bacteriostatic agent, chloramphenicol inhibits protein synthesis without directly killing cells, allowing them to remain metabolically active. Susceptible and resistant profiles can thus be distinguished in a rapid (2–3 h) ONMD test, though bacteriostatic antibiotics require the extended 180 min window. Ampicillin and ciprofloxacin suppression became more evident after 120 min, with higher concentrations producing faster signal decline.

It should be noted, however, that a classical monotonic dose-time relationship, in which higher concentrations produce progressively faster and stronger suppression at each successive time point, is not consistently observed across all panels in Figure 1, Figure 2 and Figure 3. Several factors contribute to this. First, ONMD measures single-cell mechanical activity rather than population-level growth, and the relationship between antibiotic concentration and nanomotion is not necessarily monotonic: transient stress responses, including increased cellular agitation, may precede suppression, particularly at early time points. This is most evident for chloramphenicol, where bacteriostatic inhibition of protein synthesis initially resulted in increased nanomotion before delayed suppression occurred at 180 min. Second, the tested *Komagataeibacter* species exhibit intrinsically low baseline nanomotion signal while *K. melaceti* remained resistant, limiting the dynamic range available to resolve graded concentration-dependent differences. Third, when tested concentrations substantially exceed the MIC, a ceiling effect may occur in which all supra-MIC doses produce near-complete suppression, obscuring dose-dependent gradations (e.g., ampicillin in *K. xylinus*, MIC = 0.75 µg/mL, tested up to 250 µg/mL). Conversely, under resistant conditions where none of the tested concentrations reach the MIC (e.g., ciprofloxacin in *K. melaceti*, MIC > 32 µg/mL), minimal suppression is observed regardless of dose. Finally, because different cells are analysed at each time point, stochastic single-cell variability may partially obscure dose-dependent trends, particularly when the effect sizes are modest. Despite these deviations from a classical pharmacodynamic curve, the overall pattern of nanomotion suppression in susceptible conditions and preserved activity in resistant conditions remained consistent across all tested organism–antibiotic combinations, supporting the utility of ONMD for phenotypic susceptibility classification.

A distinct mechanical phenotype was observed in motile *E. coli*. At baseline, OCP heatmaps showed discrete nanomotion puncta superimposed with elongated swimming trajectories (Figure 4d). Both confined intracellular nanomotion and translational motility contributed to the observed pixel variation.

Following antibiotic exposure, motility-associated trajectories diminished in a concentration- and time-dependent manner. At concentrations at or above the MIC, swimming traces became progressively shorter and less frequent. At higher concentrations, OCP maps transitioned from trajectory-dominated patterns to isolated puncta and eventually to globally attenuated signals (Figure 4d). This indicates suppression of translational motility accompanied by reduction in intracellular mechanical fluctuations under antibiotic stress.

ONMD distinguishes between these motion components: in motile organisms, the mechanical signature reflects both active locomotion and intracellular dynamics, whereas in non-motile bacteria it represents confined nanomechanical activity. The attenuation of swimming trajectories in *E. coli* provides a spatially interpretable indicator of physiological perturbation. Notably, the ONMD response of *E. coli* to ciprofloxacin was weaker than expected given its low MIC (0.004 µg/mL), with only −33.8% suppression at the maximum concentration. This may reflect compensatory stress responses at supra-MICs, or reduced sensitivity of OCP quantification to ciprofloxacin’s mechanism (DNA replication inhibition) compared to cell wall-disrupting agents. Concentrations closer to the MIC may yield more pronounced signals. This disparity between high susceptibility (MIC = 0.004 µg/mL) and modest ONMD suppression warrants closer mechanistic examination. Ciprofloxacin inhibits DNA gyrase and topoisomerase IV, blocking DNA replication and triggering double-strand breaks, but does not directly affect membrane integrity, existing metabolic enzymes, or the flagellar motor apparatus [18,32]. Consequently, two distinct consequences arise for the ONMD signal. First, flagellar motility, which dominates the pixel-change signal in the DiffImage analysis, depends on pre-existing flagellar complexes and proton motive force-driven motor rotation, neither of which requires de novo DNA synthesis. Cells can therefore continue swimming for extended periods after ciprofloxacin exposure, sustaining the motility-associated pixel-change component even when DNA replication has ceased. This contrasts sharply with ampicillin, which disrupts cell wall integrity and rapidly compromises both membrane potential and motility. Second, ciprofloxacin triggers the SOS response in *E. coli*, inducing expression of DNA repair enzymes and inhibiting cell division through SulA-mediated FtsZ sequestration [18,33,34]. The resulting filamentation, continued cell growth without division, produces elongated cells with potentially increased intracellular mechanical activity due to ongoing metabolism in a larger cellular volume, partially offsetting the expected nanomotion suppression. The DiffImage method, which integrates total pixel change across the entire field of view, may therefore underestimate the lethal effect of ciprofloxacin because the signal is sustained by residual flagellar motility and SOS-induced metabolic activity, even as the cells are irreversibly committed to death through chromosome fragmentation [34]. This mechanism-dependent limitation highlights that the ONMD signal is a composite readout of multiple mechanical processes, and that antibiotics targeting DNA replication may require either longer observation windows or complementary single-cell tracking to fully resolve their effect on intracellular nanomotion independent of translational motility.

These findings demonstrate that ONMD captures dynamic response trajectories rather than static endpoints, enabling discrimination between susceptible and resistant responses within short experimental timeframes.

From a food safety perspective, the high MIC values observed for *Komagataeibacter* strains are noteworthy. *K. xylinus* and *K. melaceti* both exhibited chloramphenicol MICs exceeding the highest tested concentration (>256 µg/mL), and *K. melaceti* was additionally resistant to ciprofloxacin (>32 µg/mL). These values are substantially higher than those of the *E. coli* reference strain and indicate robust intrinsic or acquired resistance phenotypes. Because kombucha is typically consumed unpasteurised and contains viable bacteria at the point of ingestion, the presence of highly resistant *Komagataeibacter* strains raises the question of whether resistance determinants could be transferred to gut-resident bacteria via horizontal gene transfer, as discussed in the Introduction [9,11]. Although *Komagataeibacter* species are generally regarded as non-pathogenic, the potential for resistance gene dissemination through the food chain warrants further investigation, particularly given the growing consumer demand for unpasteurised fermented beverages.

This study is limited by the absence of long-term adaptation measurements and by the lack of direct correlation with molecular markers of stress or metabolic state. Future work could combine ONMD with transcriptomic profiling to test whether the transient nanomotion increase observed under chloramphenicol exposure correlates with upregulation of stringent response and envelope stress genes, and whether the persistent motility signal in ciprofloxacin-treated *E. coli* reflects sustained flagellar gene expression despite SOS-mediated cell division arrest. Additionally, each strain–antibiotic combination was tested in a single experimental run, with fields of view serving as technical replicates; independent biological replicates from separate cultures were not performed. While the consistency of the observed dose–response patterns across multiple fields of view supports the reliability of the findings, independent replication would strengthen statistical confidence and is recommended for future studies. Additionally, the present study tested three antibiotics representing distinct mechanisms of action; extending the panel to include other classes to which AAB resistance has been reported, such as tetracyclines and macrolides [9,10], would provide a more comprehensive validation of ONMD-based susceptibility profiling in these organisms. In clinical antimicrobial susceptibility testing, antibiotic panels are typically tailored to Gram-positive or Gram-negative organisms; although the three antibiotics used here are all active against Gram-negative bacteria, a broader Gram-negative-oriented panel would strengthen the translational relevance of ONMD-based profiling for these organisms. Furthermore, the present work used type strains and culture collection isolates rather than clinical isolates; validation of ONMD-based susceptibility classification against clinical strains with characterised resistance profiles remains an important future direction but was beyond the objectives of this study. Similarly, the use of isogenic mutant controls to mechanistically confirm resistance pathways would complement the phenotypic observations reported here and represents a valuable avenue for subsequent investigations. Finally, although *Komagataeibacter* species are known cellulose producers, the shaken liquid culture conditions used here yielded planktonic cells not embedded in a nanocellulose matrix (see Section 2.1), and cellulose production is therefore unlikely to have confounded the nanomotion measurements in this study. The ONMD susceptibility classification thresholds used here (≥40% nanomotion suppression = susceptible; 20–40% = intermediate; <20% = resistant) were defined empirically based on the observed concordance with MIC-derived susceptibility profiles in the present study system. No standardised ONMD classification criteria currently exist in the literature: previous ONMD studies have reported statistically significant nanomotion suppression as low as 14.5% in susceptible organisms [26], but did not propose formal percentage-based breakpoints. The thresholds used here should therefore be regarded as study-specific and are not intended for direct generalisation to other organisms, antibiotics, or ONMD platforms. Unlike clinical susceptibility breakpoints (e.g., CLSI or EUCAST), which are derived from large-scale pharmacokinetic/pharmacodynamic data correlating MIC values with clinical outcomes, ONMD thresholds reflect the magnitude of mechanical signal change and are inherently dependent on the measurement platform, signal-to-noise characteristics, and baseline nanomotion of the organism under study. Establishing validated, universal ONMD susceptibility criteria would require multi-centre studies across diverse organism–antibiotic combinations with clinical outcome correlation, which is beyond the scope of the present work but represents an important direction for future research.

Overall, ONMD complements conventional susceptibility testing by resolving time-dependent suppression of both translational motility and intracellular nanomotion at the single-cell level.

Statistically significant fluctuations in the nanomotion signal of untreated control cells over time were observed across all three species and antibiotic panels. These fluctuations reflect the inherent biological variability of living cells confined in the analysis chamber, including metabolic adaptation to the microenvironment, nutrient consumption, and confinement effects. Such baseline variability does not compromise the interpretation of antibiotic-induced effects, which are distinguished by substantially larger effect sizes and a consistent dose–response relationship with increasing antibiotic concentration.

## 5. Conclusions

This study demonstrates that optical nanomotion detection (ONMD) enables rapid, time-resolved characterization of antibiotic-induced physiological perturbations in kombucha-associated acetic acid bacteria and *E. coli* at the single-cell level. By integrating conventional MIC determination with dynamic nanomotion analysis, antimicrobial susceptibility can be resolved not only as a growth endpoint but as a continuous mechanical signature reflecting metabolic disruption.

In *Komagataeibacter* species, susceptible conditions resulted in progressive suppression of confined single cell nanomotion, whereas resistant profiles maintained sustained mechanical activity. Chloramphenicol exposure initially produced resistance-like nanomotion patterns at 120 min; however, extending the observation to 180 min revealed a delayed intermediate response in *K. xylinus* and delayed susceptibility in *K. rhaeticus*, while *K. melaceti* remained resistant. Based on these observations, we recommend an extended observation window of at least 180 min when evaluating bacteriostatic antibiotics by ONMD, as the standard 120 min protocol may underestimate susceptibility due to the delayed onset of metabolic suppression characteristic of these agents. Future studies could further refine these kinetics by incorporating additional intermediate time points (e.g., 150 min) to better resolve the transition between the initial nanomotion increase and subsequent suppression observed with chloramphenicol. In motile *E. coli*, ONMD revealed a composite mechanical signal consisting of intracellular nanomotion puncta and swimming trajectories. Antibiotic exposure led to concentration- and time-dependent attenuation of motility-associated traces accompanied by reduction in intracellular mechanical fluctuations.

These findings demonstrate that ONMD complements conventional susceptibility testing by resolving distinct modes of bacterial motion and their suppression under antibiotic stress, providing dynamic single-cell insight into antimicrobial response in fermentation-relevant microbial systems. Moreover, the results reveal that certain kombucha-associated bacteria exhibit resistance to antibiotics such as ciprofloxacin and chloramphenicol, highlighting the potential presence of antimicrobial resistance traits within fermentation-associated microbiota.

## Figures and Tables

**Table 1:** Minimum inhibitory concentration (MIC) values of antibiotics against *Komagataeibacter* spp. and *E. coli* determined by MIC gradient strips.

Ampicillin	Chloramphenicol	Ciprofloxacin	MIC (µg/mL)
0.75	>256	3	*K. xylinus*
3	48	8	*K. rhaeticus*
24	>256	>32	*K. melaceti*
3	8	0.004	*E. coli*

**Table 2:** Comparison of MIC-based susceptibility classification and ONMD nanomotion response for each bacterial strain and antibiotic combination. Nanomotion change (%) represents the range of median velocity change across tested concentrations between 0 min (before treatment) and 120 min, except for *K. xylinus* and *K. rhaeticus* with chloramphenicol which compares 0 min to 180 min. For *E. coli*, nanomotion change values represent the temporal response at the highest tested concentration (at 10, 60, and 120 min versus the untreated control at the same timepoint), whereas *p*-values are reported per concentration at 120 min. Negative values indicate a decline in nanomotion; positive values indicate an increase. ONMD susceptibility classification was based on the magnitude of nanomotion suppression relative to baseline (120 min for most conditions; 180 min for chloramphenicol-treated *K. xylinus* and *K. rhaeticus*): strong suppression (>40%) indicates susceptibility (classified as “Susceptible”), weak suppression (<20%) indicates resistance, and intermediate responses (20–40%) represent partial physiological perturbation. A “delayed” label denotes cases where initial resistance-like behavior at 120 min shifted toward suppression by 180 min: “Susceptible (delayed)” is applied when suppression exceeds 40% at one or more concentrations by 180 min, and “Intermediate (delayed)” when suppression remains within the 20–40% range. These thresholds were defined empirically for the present study system and are not intended as universal ONMD classification criteria; different organisms or experimental conditions may require adapted threshold values. Statistical significance (Mann–Whitney U test for *Komagataeibacter* spp.; Welch’s *t*-test for *E. coli*) is shown per concentration (c_1_/c_2_/c_3_ = MIC/supra-MIC/maximum). * *p* < 0.05, *** *p* < 0.001, **** *p* < 0.0001; ns, not significant.

		Ampicillin	Chloramphenicol	Ciprofloxacin
*K. xylinus*	MIC strip	0.75	>256	3
	MIC classification	Susceptible	Resistant	Susceptible
	Nanomotion change (c_1_/c_2_/c_3_) (%)	−38.4/−63.8/−26.8	−34.4/−20.4/−34.9	−17.0/−57.5/−42.9
	*p* value (c_1_/c_2_/c_3_)	****/****/****	****/****/****	****/****/****
	ONMD classification	Susceptible	Intermediate (delayed)	Susceptible
*K. rhaeticus*	MIC strip	3	48	8
	MIC classification	Susceptible	Susceptible	Susceptible
	Nanomotion change (c_1_/c_2_/c_3_) (%)	−32.1/−43.5/−35.6	7.3/7.4/−43.8	−51.5/−70.1/−48.3
	*p* value (c_1_/c_2_/c_3_)	****/****/****	*/*/****	****/****/****
	ONMD classification	Susceptible	Susceptible (delayed)	Susceptible
*K. melaceti*	MIC strip	24	>256	>32
	MIC classification	Susceptible	Resistant	Resistant
	Nanomotion change (c_1_/c_2_/c_3_) (%)	−35.5/−56.9/−38.6	63.3/47.0/33.5	−9.8/−18.7/−4.8
	*p* value (c_1_/c_2_/c_3_)	****/****/****	****/****/****	*/****/ns
	ONMD classification	Susceptible	Resistant	Resistance/weak physiological response
*E. coli*	MIC strip	3	8	0.004
	MIC classification	Susceptible	Susceptible	Susceptible
	Nanomotion change (%)	−49.7/−69.5/−88.3	−44.2/18.0/−72.3	−1.7/4.0/−33.8
	*p* value (c_1_/c_2_/c_3_)	***/***/***	*/*/*	ns/*/ns
	ONMD classification	Susceptible	Susceptible	Susceptible

## Data Availability

All data needed to evaluate the conclusions in the paper are present in the paper. Additional data related to this paper may be requested from the authors. A precompiled Windows executable of the PyONMD version 2 analysis software (“PyONMD_Ana_02.exe”) is available upon request.

## Abbreviations

The following abbreviations are used in this manuscript:

ONMD	Optical nanomotion detection
BNC	Bacterial nanocellulose
MIC	Minimum inhibitory concentration
AMR	Antimicrobial resistance
AAB	Acetic acid bacteria
OCP	Optical changing pixels
FOV	Field of view
